# Risk of Colorectal Cancer in Patients With Irritable Bowel Syndrome: A Meta-Analysis of Population-Based Observational Studies

**DOI:** 10.3389/fmed.2022.819122

**Published:** 2022-03-02

**Authors:** Xinhui Wu, Jingxi Wang, Zhen Ye, Jin Wang, Xibei Liao, Mengsi Liv, Zhen Svn

**Affiliations:** ^1^Hospital of Chengdu University of Traditional Chinese Medicine, Chengdu, China; ^2^Stomatological Hospital of Chongqing Medical University, Chongqing Medical University, Chongqing, China; ^3^Hengyang Medical School, University of South China, Hengyang, China

**Keywords:** irritable bowel syndrome, colorectal cancer, epidemiology, CRC screening, meta-analysis, risk factor

## Abstract

**Background and Aims:**

Evidence on the association between irritable bowel syndrome (IBS) and colorectal cancer (CRC) risk is inconsistent. Therefore, we aimed to examine whether IBS leads to an increased risk for CRC using a systematic review and meta-analysis approach.

**Methods:**

PubMed, Embase, and Web of Science were systematically searched to identify all relevant literature published through July 30, 2021. The pooled risk ratios (RRs) and corresponding 95% confidence intervals (CIs) for CRC after diagnosis of IBS were computed using random-and fixed-effects models and stratified by age, follow-up time, gender, and study design. The quality of included studies was assessed by the Newcastle-Ottawa scale.

**Results:**

We included six studies consisting of 1,085,024 participants. Overall, the risk of detecting CRC after the initial IBS diagnosis was significantly higher than non-IBS controls (RR = 1.52, 95% CI: 1.04–2.22, *P* = 0.032). The peak of elevated risk occurred within the first year of IBS diagnosis (RR = 6.84, 95% CI: 3.70–12.65, *P* < 0.001), and after 1 year, the risk of CRC was similar to that of the general population (RR = 1.02, 95% CI: 0.88–1.18, *P* = 0.813). Notably, we found that the RR of CRC was more significant in IBS patients younger than 50 years compared to those older than 50 years (RR = 2.03, 95% CI: 1.17-3.53, *P* = 0.012 vs. 1.28, 95%CI: 0.94-1.75, *P* = 0.118, respectively). Gender and study design did not affect the results.

**Conclusion:**

The risk of CRC within one year of the initial IBS diagnosis was increased approximately six-fold, whereas the long-term risk was not increased. However, current evidence does not support that IBS leads to an increased incidence of CRC, and the early excess risk is more likely attributable to misclassification resulting from overlapping symptoms rather than causation. Clinicians must remain vigilant for the CRC risk in patients younger than 50 years with IBS-like symptoms to avoid delaying necessary screening.

## Introduction

Irritable bowel syndrome (IBS) is one of the leading functional gastrointestinal disorders, affecting more than 9% of the global adults according to the Rome III criteria (1). Traditionally, IBS is considered to be free of biochemical or structural abnormalities and is characterized clinically by chronic or recurrent abdominal pain, discomfort, bloating, and altered bowel habits (2). IBS has similar symptoms with various organic gastrointestinal conditions, and distinguishing it from colorectal cancer (CRC) is crucial (3, 4). CRC is the third most common cancer globally, causing nearly 700,000 deaths worldwide each year (5). Patients with IBS are primarily concerned and anxious about their potential risk of developing CRC (6).

In recent years, multiple factors associated with IBS development, including low-grade mucosal inflammation, immune activation disorders, altered intestinal microbiota, neuroendocrine system disorders, and metabolic abnormalities, have been elucidated. IBS does not seem to be a purely functional disease, so it may be reasonable to hypothesize that IBS can introduce certain risk factors that promote tumorigenesis (7–9). For example, there is growing evidence that inflammation and tumorigenesis are related (10–13). Moreover, there may be common exposures between IBS and CRC (14, 15). Although some studies found that the yield of colonoscopy showed no meaningful difference in the prevalence of CRC between IBS patients and non-IBS controls (16, 17), inconsistent results have emerged from population-based studies that explored the association between IBS and subsequent CRC risk (18, 19).

Both IBS and CRC place a huge burden on global health systems and economies; due to the high prevalence of IBS, any association with CRC risk will have essential impacts on clinical practice. Given the current inconsistent evidence, we conducted this meta-analysis to examine the association between IBS and subsequent CRC risk, taking into account age, sex, follow-up time, and study design.

## Materials and Methods

We reported this systematic review and meta-analysis based on the Preferred Reporting Items for Systematic Reviews and Meta-Analyses guidelines (20). The protocol for this study is not registered.

### Literature Search

A systematic search was conducted in Embase, PubMed, and Web of Science to identify all relevant literature published from database inception to July 30, 2021, without language restrictions. The search strategy, developed from Medical Subject Headings combined with synonyms, included (“irritable bowel syndrome” OR “IBS” OR “irritable colon”) and (“colorectal cancer” OR “CRC” OR “colorectal neoplasms” OR “colorectal tumor” OR “colorectal carcinoma”). Further details of the search strategy for each database are provided in the Supplementary Materials. No filters were used during the search. In addition, we manually searched the reference lists of included studies and relevant reviews to avoid omitting additional appropriate studies.

### Selection Criteria

Two reviewers independently screened the titles/abstracts and full-text of initial search results. Eligible records were original studies published as full articles that explored the risk of CRC occurrence after IBS diagnosis and reported the risk ratio (RR) or standardized incidence ratio (SIR) along with corresponding 95% confidence intervals (CIs) compared to the non-IBS cohort or expected number. The PICOS elements of research question are presented in Table 1. We excluded studies that did not establish a temporal relationship between IBS and CRC, such as cross-sectional studies. Case reports and non-human studies would be excluded. If multiple reports were from the same population and the study methods were identical, only the most complete report would be included in the analysis. If multiple reported patients did not completely overlap and there were differences in study methodology, all were included in the systematic review, and sensitivity analyses would be performed subsequently to assess the impact of potentially overlapping data on the pooled result.

**Table 1:** The PICOS criteria for the definition of research question.

Populations	Non-specific
Intervention	Patients diagnosed with irritable bowel syndrome
Comparator	Patients without a diagnosis of irritable bowel syndrome, general population
Outcome	Risk ratio or standardized incidence ratio for colorectal cancer
Study design	Cohort study, case-control study

### Data Extraction and Quality Assessment

Two reviewers screened full-text based on inclusion/exclusion criteria and separately extracted data from eligible studies. Any disagreements were resolved by agreement among all authors. The following information was extracted from each study: first author's name, year of publication, region, study design, study period, sex, age, population source, sample size of the observed cohort, methods of diagnosis of IBS and CRC, matched/adjusted/standardized confounding factor, follow-up time, and risk estimates along with corresponding 95% CI.

The Newcastle-Ottawa scale (NOS) was used to assess the quality of included studies in terms of selection, comparability, and outcome (21). The study with NOS scores greater than six was considered high quality; otherwise, it was considered to be at high risk of bias.

### Statistical Analyses

The primary endpoint of this study was the pooled risk estimate of CRC occurrence after the initial diagnosis of IBS. If a study provided crude and adjusted risk estimates, adjusted risk estimates were preferentially included in the analysis. Heterogeneity between included studies was assessed by Cochran's Q-test and Higgins' I^2^ statistics. When I^2^ ≥ 50% or *P* ≤ 0.1, substantial heterogeneity was considered to exist, and the random-effects model was used to compute the pooled risk estimates; when I^2^ <50% and *P* > 0.1, heterogeneity was considered mild, and the fixed-effects model was applied. We looked for possible sources of heterogeneity by reviewing the characteristics of included studies. The stability of the analyses was tested by excluding one study at a time and then repeating the meta-analysis and was further verified by comparing the pooled results of the random- and fixed-effects models. Publication bias was assessed by funnel plots and confirmed by Begg's and Egger's tests. If the included studies were symmetrically distributed in the funnel plot and the *P*-values of both the Begg' and Egger' tests were >0.05, no publication bias was considered to exist; otherwise, the impact of potential publication bias was assessed by the trim-and-fill method. Of note, funnel plots were omitted if fewer than 10 studies were finally included. Moreover, we explored the impacts of follow-up time (≤1 vs. >1 year), age (<50 vs. ≥50 years), gender (female vs. male), and study design (prospective vs. retrospective) on risk estimates by subgroup analyses. STATA/MP 16.0 was used to perform data analysis in the study. All *P*-values were two-tailed, and the threshold value for statistical significance is below 0.05.

## Results

**Figure 1:**
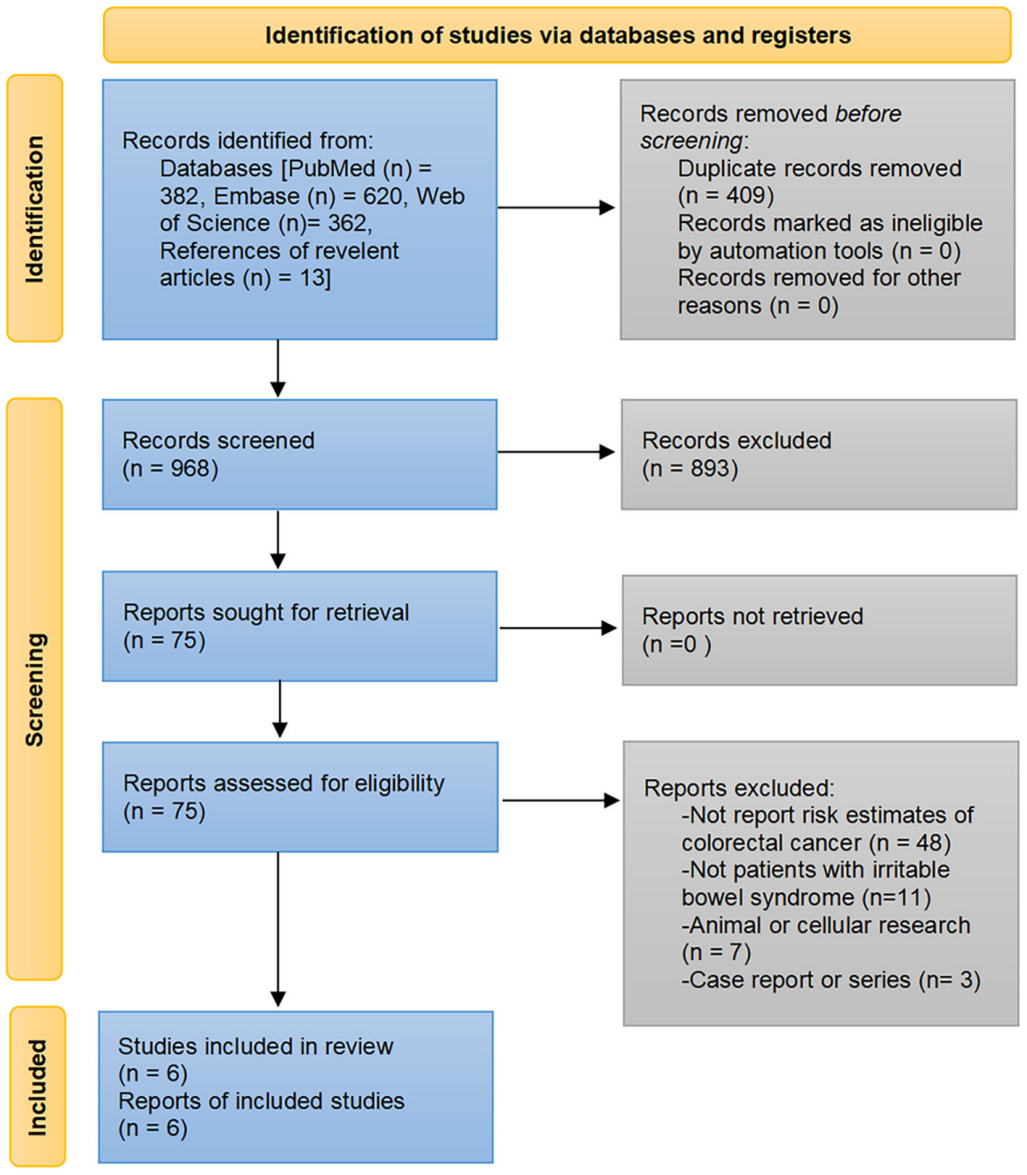
Flowchart of study selection.

Of the 1,364 publications initially searched from PubMed, Embase, and Web of Science databases and the additional 13 articles identified through manual review of the bibliography, six studies comprising 1,085,024 participants met the eligibility criteria to be included for analysis (18, 19, 22–25). The detailed process of study selection and reasons for exclusion are presented in Figure 1.

The included studies were conducted in three regions: China (18, 24, 25), the United Kingdom (22, 23), and Denmark (19). All included studies were population-based cohort studies with sample sizes ranging from 39,384 to 659,757. Except for one study in which CRC was determined by the diagnosis of colorectal colonoscopy (18), patients with IBS and CRC in the other studies were identified by diagnostic codes from the health insurance registry or clinical research database, such as the International Classification of Diseases codes. Four studies evaluated the CRC risk in the IBS cohort compared to the non-IBS cohort (18, 22–24), and two studies computed standardized incidence ratios (SIRs) for the IBS cohort as a measure of risk effects by comparing the observed number of CRC with the expected number (19, 25). Both studies from the United Kingdom used the same database, but the study period and the number of participants in the study conducted by Canavan et al. were greater than those in Rodriguez et al. (22, 23). Therefore, only the study by Canavan et al. was included in our quantitative meta-analysis, and the study by Rodriguez et al. was analyzed qualitatively only. Two studies from the Taiwanese population used the Health Insurance Database, but they included patients differently and used different analysis methods (24, 25). In the study by Chang et al. the IBS patients were obtained from National Health Insurance, but the CRC was not identified in the same way and the study methods were different from the other two (18); therefore, all three studies from Taiwan were included in the meta-analysis. There were three retrospective and three prospective studies with a median/mean follow-up of 3.0–8.8 years, and five studies with a maximum follow-up of more than 10 years (18, 19, 22, 24, 25). Detailed information on the characteristics of the included studies is presented in Table 2.

**Table 2:** Main characteristics of the eligible studies.

**References**	**Location**	**Study period**	**Study design**	**Age-yr**.	**Gender (F/M)**	**Population**	**IBS**	**Non-IBS**	**Identification of IBS**	**Identification of CRC**	**Confounding factors considered**	**Followed up-yr**.
Chang et al. (18)	Taiwan, China	1999–2009	Prospective cohort	Mean 55.9	23,847/15,537	Community	9,160	30,224	ICD code	Colonoscopy and national cancer registry data	Adjusted for age, gender, BMI, CRC family history, alcohol drinking, education, diabetes, and hypertension	Mean 7.78
Canavan et al. (22)	UK	Since 1987	Prospective cohort	IBS: mean 42.9; non-IBS: mean 42.8	476,900/182,857	UK CPRD	112,854	546,903	Read code	CRC Read term code or ICD code	Matched by sex and age	IBS: mean 6.5; non-IBS: mean 3.6
García Rodriguez et al. (23)	UK	1994–1998	Prospective cohort	20–79	NP	England and Wales GPRD	2,956	20,000	Read code	Read code	Adjusted for age and gender	Mean 3
Nørgaard et al. (19)	Denmark	1977–2008	Retrospective cohort	Median 47	39,998/17,853	DNRP	57,851	Expected incidence rate	ICD code	ICD code	Standardized on period of follow-up, age, gender, and time of diagnosis	Mean 8.8
Hsiao et al. (24)	Taiwan, China	2000–2010	Retrospective cohort	IBS: mean 44.6; non-IBS: mean 44.4	148,878/126,360	LHID	91,746	183,492	ICD code	ICD code	Matched by age, gender, and time of diagnosis; adjusted for age, gender, urbanization, hypertension, diabetes, and hyperlipidemia	1–11
Hu et al. (25)	Taiwan, China	2000–2010	Retrospective cohort	Median 50.9	13,727/16,111	NHIRD	29,838	Expected incidence rate	ICD code (≥3 visits and diagnosis was not altered within 3 months)	Registry for Catastrophic Illness (histologic confirmation is required)	Standardized on age, gender, and duration of IBS	Median 4.56

*IBS, irritable bowel syndrome; CRC, colorectal cancer; ICD, International Classification of Diseases; F/M, female/male; NP, not provided; CRPD, Clinical Practice Research Datalink; GRPD, General Practice Research Database; DNRK, Danish National Registry of Patients; LHID, Longitudinal Health Insurance Database; NHIRD, Taiwan National Health Insurance Research Database*.

The NOS score was 7 for four studies and 8 for the other two, indicating that the overall quality of the included studies was high. The main risk of bias was the inability to demonstrate that CRC was not present at the start of their study (Table 3).

**Table 3:** The quality assessment of included studies.

**Study**	**Representativeness of exposed cohort**	**Selection of non-exposed cohort**	**Ascertainment of exposure**	**Outcome not present before study**	**Comparability**	**Assessment of outcome**	Follow-up long enough^*^	**Adequacy of follow up**	**Quality score**
Chang et al. (18)	⋆	⋆	⋆	✰	⋆⋆	⋆	⋆	⋆	8
Canavan et al. (22)	⋆	⋆	⋆	✰	⋆✰	⋆	⋆	⋆	7
García Rodriguez et al. (23)	⋆	⋆	⋆	✰	⋆⋆	⋆	✰	⋆	7
Nørgaard et al. (19)	⋆	⋆	⋆	✰	⋆✰	⋆	⋆	⋆	7
Hiso et al. (24)	⋆	⋆	⋆	✰	⋆⋆	⋆	⋆	⋆	8
Hu et al. (25)	⋆	⋆	⋆	✰	⋆✰	⋆	⋆	⋆	7

* *A median/mean follow-up of more than 3 years or a maximum follow-up of more than 10 years was considered adequate*.

### Association Between Initial Diagnosis of IBS and Subsequent Risk of CRC

**Figure 2:**
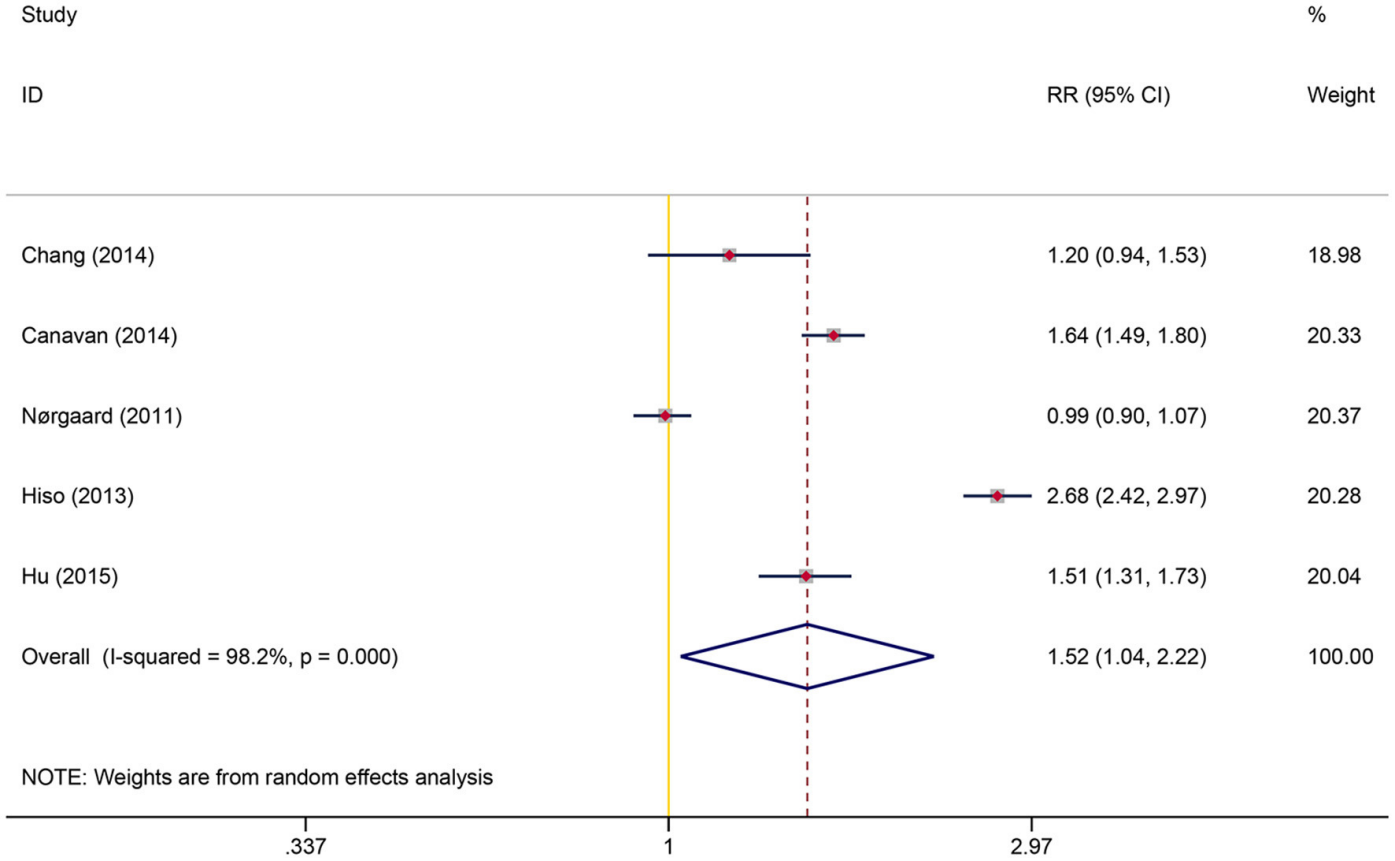
Overall association between irritable bowel syndrome and subsequent colorectal cancer risk.

A total of five studies involving 1,062,068 participants were included in the overall meta-analysis; heterogeneity testing indicated substantial heterogeneity among studies (I^2^ = 98.2%, *P* < 0.001), so the random-effects model was applied. The pooled result showed a significantly increased risk of detecting CRC following IBS diagnosis than controls (RR = 1.52, 95% CI: 1.04–2.22, *P* = 0.032) (Figure 2). After reviewing the study characteristics, we did not find a clear source of heterogeneity, probably due to inconsistencies in clinicians' views on diagnosing IBS across different regions and in the definition of IBS at different periods.

Stratified analyses were then performed to explore the effects of follow-up time, age, gender, and study design on this association. Subgroup analyses showed that the excess risk of CRC was concentrated in the first year after the diagnosis of IBS, and CRC had the highest RR of 6.84 (95% CI: 3.70–12.65, *P* < 0.001) within this period; after excluding the first year following IBS diagnosis, the RR for CRC decreased to 1.02 (95% CI: 0.88–1.18, *P* = 0.813). The study conducted by Rodriguez et al. also presented consistent findings (23). A significantly increased RR of CRC was observed in patients with IBS aged under 50 years (RR = 2.03, 95% CI: 1.17–3.53, *P* = 0.012), while no statistical significance was observed in those aged over 50 years (RR = 1.28, 95%CI: 0.94–1.75, *P* = 0.118). The association between IBS and CRC risk was similar in prospective and retrospective studies but was only statistically significant in the former. Gender did not affect CRC risk (Table 4).

**Table 4:** Subgroup analysis of the association between IBS and subsequent CRC risk.

**Subgroup**	**No. of studies**	**Risk ratio**	**95% CI**	***P*_overall effect_**	***I*^2^ static**	***P*_heterogeneity_**
*Total*	5	1.52	1.04–2.22	0.032	98.2%	<0.001
*Followed-up*						
≤ 1 year	3	6.84	3.70–12.65	<0.001	95.6%	<0.001
>1 year	4	1.02	0.88–1.18	0.813	77.1%	<0.001
*Age*						
<50 years	3	2.03	1.17–3.53	0.012	89.8%	<0.001
≥ 50 years	3	1.28	0.94–1.75	0.118	95.6%	<0.001
*Gender*						
Female	3	1.30	0.81–2.08	0.280	96.8%	<0.001
Male	3	1.32	0.71–2.46	0.376	97.6%	<0.001
*Study design*						
Prospective	2	1.43	1.06–1.94	0.020	81.8%	0.019
Retrospective	3	1.59	0.84–3.00	0.154	99.1%	<0.001

### Publication Bias

Funnel plots were not performed because included studies were less than ten. The *P*-values for Begg's and Egger's tests were 1.00 and 0.933, respectively, indicating no potential publication bias for the current study.

### Sensitivity Analysis

**Figure 3:**
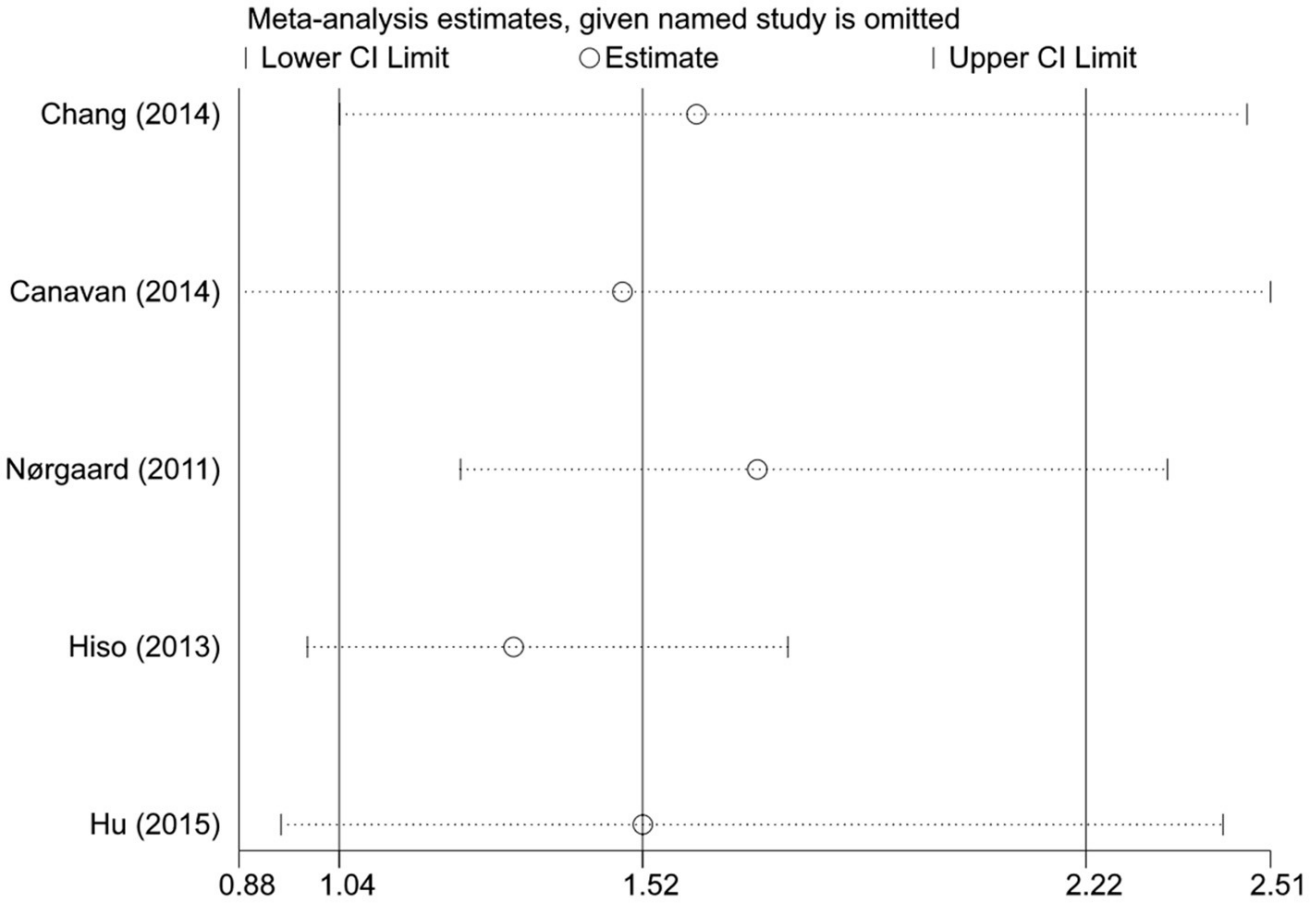
Sensitivity analysis of the colorectal cancer risk in patients with irritable bowel syndrome.

When the stability of the results was tested by excluding one cohort at a time, we found that the statistical significance of the overall association, age <50 years group, and the prospective study group disappeared when certain cohorts were excluded, but the trend of increased risk for CRC after IBS diagnosis remained unchanged (Figure 3). The pooled results of the random- and fixed-effects models were similar for each group. Due to high heterogeneity, we reported more conservative results using the random-effects model in the *results* section, whereas the pooled results of the fixed-effects model suggested that statistical correlations between IBS and CRC were present in all subgroups except for those with follow-up >1 year (Table 5).

**Table 5:** Comparison of the use of random-effects vs. fixed-effects models.

**Analysis groups**	**HR (95% CI), random-effects model**	**HR (95% CI), fixed-effects model**
Overall	1.52 (1.04–2.22)	1.52 (1.45–1.60)
Followed-up ≤ 1 year	6.84 (3.70–12.65)	6.26 (5.60–7.01)
Followed-up >1 year	1.02 (0.88–1.18)	0.98 (0.92–1.04)
Age <50 years	2.03 (1.17–3.53)	1.75 (1.48–2.06)
Age ≥ 50 years	1.28 (0.94–1.75)	1.50 (1.41–1.60)
Female	1.30 (0.81–2.08)	1.41 (1.30–1.52)
Male	1.32 (0.71–2.46)	1.72 (1.57–1.89)
Prospective	1.43 (1.06–1.94)	1.57 (1.44–1.72)
Retrospective	1.59 (0.84–3.00)	1.50 (1.41–1.59)

## Discussion

To our knowledge, this is the first systematic review and meta-analysis focused on examining the association between IBS and CRC risk. The pooled result involving more than one million participants showed a 52% increased risk of detecting CRC after the IBS diagnosis. However, when stratified based on follow-up time, the increased risk of CRC was concentrated only in the first year following IBS diagnosis, and the excess risk disappeared after 1 year. Patients with IBS under 50 years seemed to have a higher relative risk than those aged more than 50 years. Gender did not affect the correlation between IBS and CRC. The pooled result from the retrospective studies was not statistically significant, perhaps due to the small number of included studies and thus lack of statistical power.

Although the data suggest that patients have a higher incidence of CRC after IBS diagnosis than non-IBS controls, it does not demonstrate that the increased risk can be attributed to IBS. CRC is characterized by a long latency period, so if IBS can cause tumorigenesis by certain mechanisms, the risk of CRC would increase with prolonged follow-up (26, 27). However, the increased risk is only observed in the first year of the initial IBS diagnosis. Furthermore, if IBS and CRC share common exposure factors, the high prevalence of CRC in patients with IBS should also continue to increase. Therefore, the current results more support the misclassification of CRC. Because IBS and CRC have overlapping symptoms, especially during mild disease activity, for example, both may present with abdominal pain and change in bowel habits; therefore, patients with CRC-related symptoms were initially misinterpreted as IBS (4). Hence, the ICD codes used in the included studies to identify IBS may not represent a final diagnosis, and patients with IBS-like symptoms were not correctly diagnosed until they exhibited alarm symptoms as CRC progressed. Patients with IBS were also more likely to detect CRC due to more frequent surveillance. In addition, two studies from Taiwan and the United Kingdom found that the IBS cohort had a higher relative risk of subsequently detecting colorectal adenomas than CRC, which also supports the explanation that CRC was initially misdiagnosed as IBS (18, 23); as adenomas often show milder gastrointestinal symptoms than cancers, making them more likely to be misdiagnosed as IBS.

Of note, a concerning finding is that patients younger than 50 had more than twice the risk of detecting CRC after the initial IBS diagnosis than the general population and even had higher relative risk than patients older than 50. As discussed above, if the increased risk of CRC in patients with IBS is mainly attributable to misdiagnosis, it implies that the probability of misdiagnosis is higher in those under age 50. The current international guideline for the IBS diagnosis, the Rome IV criteria, recommends that patients with IBS undergo colonoscopy for evaluation of organic bowel diseases only if they present with alarm symptoms such as bloody stools, unintended weight loss, unexplained iron deficiency anemia, nocturnal symptoms, abdominal masses or lymph node enlargement, fever, family history of CRC, and age of onset >50 years (28). Some expert consensus also does not recommend colonoscopy for patients younger than 50 with IBS-like symptoms without alarm symptoms (29, 30). However, pooled data from the current studies suggest that patients under 50 years appeared to be more likely to miss a diagnosis of CRC at the time of their initial visit, which may mean that physicians are not sufficiently alert to CRC when dealing with younger patients. The incidence of CRC increases with age, especially in the fifth decade of life, which is why most guidelines recommend screening programs only for those over 50 years of age in the absence of alarm symptoms (5, 31). With the introduction of aggressive screening and education about CRC, the overall incidence of CRC has been declining in the United States. However, this declining trend has occurred primarily in older adults; since 2012, the annual incidence of CRC has increased by two percent among those younger than 50 years, particularly among those aged 40–44 years (32, 33).

Early-onset CRC has attracted increasing attention from researchers in recent years, and reports from different regions around the world confirmed that the proportion of early-onset CRC is continuing to rise (34–38). Patients with early-onset CRC have a longer delay from symptom onset to diagnosis and are diagnosed at a higher rate of advanced stages than older patients (39, 40). CRC is relatively slow to develop, and aggressive screening may reduce the incidence and allow for diagnosis before it progresses to an incurable stage, thereby reducing mortality and prolonging life (41, 42). Some studies have analyzed the feasibility of lowering the age for CRC screening, and the American Cancer Society recommends initiating screening at age 45 instead of 50 for adults at average risk (43–46). Our study suggests that lowering the recommended screening age for IBS patients or establishing a lower recommended screening threshold for IBS patients under 50 years may also benefit early diagnosis. Primary care physicians must be alert to the risk of CRC in young patients with IBS-like symptoms. In areas with constrained medical resources, unnecessary referrals and invasive tests may result in health and financial burdens, so alternative, less costly modalities such as more detailed medial history taking, stool tests, radiologic tests, and blood tests can be considered (47, 48).

An interesting finding from the Danish population study was the significantly decreased long-term risk of CRC in patients who received a colonoscopy or flexible sigmoidoscopy 3 months before or after their first recorded IBS diagnosis. After stratifying according to follow-up time, the proportion of these IBS patients with CRC detected within 3 months was also higher than in overall IBS patients, which could be explained by the earlier detection of CRC due to using colonoscopy, as the presumptive diagnosis of IBS that was initially recorded was not corrected after the confirmatory diagnosis of CRC. In addition, studies including multiple randomized controlled trials have found that colonoscopy or sigmoidoscopy screening could reduce long-term incidence and mortality of CRC. This study from Denmark showed that this protective effect of screening also appears to be present in patients with IBS (19, 49–53).

The main limitation of this study is the relatively small number of included studies, and therefore the pooled results in some groups are not robust enough. Second, although the long follow-up time allowed assessment of the long-term effects of IBS, the definition of IBS may change during the study period. In addition, it is still controversial whether to use a positive symptom-based or an exclusionary diagnostic strategy for IBS. These may account for the high heterogeneity among studies. Third, the included studies could only guarantee a temporal relationship between IBS and CRC regarding diagnosis rather than onset time. Therefore, no causal relationship can be inferred. Fourth, due to the nature of observational studies, some potential confounding variables such as smoking habits, alcohol consumption, physical activity, and diet were not measured or adjusted for in the included studies. Finally, whether the association between different IBS subtypes and colon or rectal cancers differed was not analyzed due to lack of data.

## Conclusion

Patients with IBS do not have an increased risk of long-term CRC development. The dramatically higher risk of CRC within the first year after IBS diagnosis may be attributed to misdiagnosis resulting from overlapping symptoms between the two diseases. It is important to note that this risk is higher in people younger than 50 years than those older than 50 years. As CRC incidence continues to rise in younger populations, we suggest that clinicians remain vigilant for the risk of CRC and consider endoscopy in more patients when managing those under 50 years with IBS-like symptoms. Future studies exploring the feasibility of lowering the age of CRC screening in patients with IBS are warranted.

## Data Availability Statement

The original contributions presented in the study are included in the article/Supplementary Materials, further inquiries can be directed to the corresponding author.

## Author Contributions

ZS: conception and design, development of methodology, critical review and revision of the manuscript, and study supervision. ML: conception and design. ML and JingW: acquisition of data. ZY and JinW: statistical analysis. XW, JingW, ML, XL, and ZY: drafting of manuscript. All authors contributed to the article and approved the submitted version.

## Conflict of Interest

The authors declare that the research was conducted in the absence of any commercial or financial relationships that could be construed as a potential conflict of interest.

## Publisher's Note

All claims expressed in this article are solely those of the authors and do not necessarily represent those of their affiliated organizations, or those of the publisher, the editors and the reviewers. Any product that may be evaluated in this article, or claim that may be made by its manufacturer, is not guaranteed or endorsed by the publisher.
