# Supplementary material for: Risk of Colorectal Cancer in Patients With Irritable Bowel Syndrome: A Meta-Analysis of Population-Based Observational Studies
Source: Front Med (Lausanne). 2022 Mar 2;9:819122. doi: 10.3389/fmed.2022.819122 (PMC8924657; doi:10.3389/fmed.2022.819122)
Supplement: Supplementary file 1 [file Data_Sheet_1.DOCX]

**Search strategy for each database:**

**PubMed:**

("Irritable Bowel Syndrome"[Mesh] or irritable bowel syndromes or Syndrome, Irritable Bowel or Syndromes, Irritable Bowel or Colon, Irritable or Irritable Colon or Colitis, Mucous or Colitides, Mucous or Mucous Colitides or Mucous Colitis) AND ("Colorectal Neoplasms"[Mesh] or Colorectal Neoplasm or Neoplasm, Colorectal or Neoplasms, Colorectal or Colorectal Tumors or Colorectal Tumor or Tumor, Colorectal or Tumors, Colorectal or Colorectal Cancer or Cancer, Colorectal or Cancers, Colorectal or Colorectal Cancers or Colorectal Carcinoma or Carcinoma, Colorectal or Carcinomas, Colorectal or Colorectal Carcinomas)

**Embase:**

('irritable colon'/exp OR 'colon spasm':ab,ti OR 'colon, irritable':ab,ti OR 'colonic diseases, functional':ab,ti OR 'colonospasm':ab,ti OR 'functional colonic diseases':ab,ti OR 'irritable bowel syndrome':ab,ti OR 'irritable colon syndrome':ab,ti OR 'mucomembraneous colitis':ab,ti OR 'mucomembranous colitis':ab,ti OR 'mucous colitis':ab,ti OR 'spastic colitis':ab,ti OR 'spastic colon':ab,ti OR 'unstable colon') AND ( 'colorectal cancer'/exp OR 'colorectal tumor'/exp OR 'colorectal neoplasia':ab,ti OR 'colorectal neoplasm':ab,ti OR 'colorectal neoplasms':ab,ti OR 'colorectal tumour':ab,ti OR 'tumor, colorectal':ab,ti OR 'tumour, colorectal')

**Web of Science:**

TS=((Irritable Bowel Syndrome or irritable bowel syndromes or Syndrome, Irritable Bowel or Syndromes, Irritable Bowel or Colon, Irritable or Irritable Colon or Colitis, Mucous or Colitides, Mucous or Mucous Colitides or Mucous Colitis) AND (colorectal cancer or colorectal tumor or Colorectal Neoplasms or Colorectal Neoplasm or Neoplasm, Colorectal or Neoplasms, Colorectal or Colorectal Tumors or Colorectal Tumor or Tumor, Colorectal or Tumors, Colorectal or Colorectal Cancer or Cancer, Colorectal or Cancers, Colorectal or Colorectal Cancers or Colorectal Carcinoma or Carcinoma, Colorectal or Carcinomas, Colorectal or Colorectal Carcinomas))
